# Supplementary material for: Self-organization of signal transduction
Source: F1000Res. 2013 Apr 23;2:116. [Version 1] doi: 10.12688/f1000research.2-116.v1 (PMC4257141; doi:10.12688/f1000research.2-116.v1)
Supplement: Peer review history

# Peer review: Referee response for version 1

**DOI:** 10.5256/f1000research.1276.r3996

**Published:** 2014-06-03

**Authors:** Eugene Koonin

**Affiliations:** 1 National Center for Biotechnology Information, National Institutes of Health, Bethesda, MD, USA

This article reports a new approach to modeling biological signal transduction systems based on the simple but important assumption that such systems have been evolutionarily optimized for maximum signal transmission efficiency. By embedding evolutionary optimization into the equations, the author obtains a self-organizing, evolving model. The approach is not fundamentally novel as the idea comes from the well-developed field of flux balance analysis. Apparently, however, this methodology has not been previously applied to signal transduction.

The current article is somewhat too abstract to judge on the ultimate utility of the approach but certainly, this is a step in an interesting direction.

I have read this submission. I believe that I have an appropriate level of expertise to confirm that it is of an acceptable scientific standard.

# Peer review: Referee response for version 1

**DOI:** 10.5256/f1000research.1276.r2347

**Published:** 2013-11-06

**Authors:** Reimer Kühn

**Affiliations:** 1 Department of Mathematics, King's College London, London, UK

In this paper the author studies optimization of parameters in a network of bio-chemical reactions for signal-transduction in a one-input-one-output setting, seeking either to maximize a stationary response coefficient, or to maximize the slope at half-maximum of the (sigmoid) stationary response function, or to minimize the delay to steady state. Combinations of these individual criteria are also looked at. Optimization is performed over the space of kinetic rates and the initial concentrations of reactants of the set of kinetic equations making up the signal transduction system.

The paper is very interesting and addresses a valid problem. It takes a well tested tenet as its starting point, viz. that signal transduction - like many other biological processes - “has been optimized by evolution, and that concentration adjustment exists to maintain effective signal transmission.”

Thus parameters are sought which would optimize a given signal transduction system. The example studied in the present paper consists of a set of 23 chemical reactions involving 27 molecular species, and one or a combination of several criteria as mentioned above are used to drive the optimization. This paradigm, it is argued would naturally give rise to a notion of self-organization of said system, as well as support adaptability under changing external conditions.

While I find this an attractive proposition in principle, and one that could carry quite far in further attempts to understand cellular processes, I am not entirely convinced that the way in which it is implemented in the present paper is the most appropriate.

My main concern is related to the fact that, kinetic coefficients are included in the space of parameters over which optimization of response coefficients is performed. Kinetic coefficients are, however, largely given by the chemistry, or rather the physical properties of reactants involved. There is thus only a restricted scope for varying these parameters; they may be varied to be sure (though not independently!) by changing temperature, or the pH or the salinity of the solute in which these reactions occur. Since neither of these conditions would change widely under natural in-vivo conditions, there is thus a question whether this form of optimization is actually a good model of the one chosen by nature in the evolutionary process. Differences between optimal and original values of kinetic coefficients are not reported in the paper. It would be interesting to know whether they are small (hence whether nature has exploited the given reactions to the full).

Indeed, using minor modifications of the present setup, which would allow to describe the co-variation of kinetic coefficients with temperature, pH and salinity, one might thus conceivably be able to verify that - given the set of reactions describing the signalling system - nature is indeed running them under optimal conditions.

An altogether more ambitious project would consist in implementing a more appropriate model of the optimization process likely to have been implemented by nature to find precisely these 23 reactions. This form of optimization would require something of a more combinatorial nature (changing or modifying reac-

tants thus reactions themselves) rather than parameters describing given reactions. Genetic algorithms, in some sense also implemented by nature, could be the method of choice to go about it.

I have read this submission. I believe that I have an appropriate level of expertise to confirm that it is of an acceptable scientific standard.

# Response

**Published:** 2014-06-14

**Authors:** Gabriele Scheler

**Affiliations:** Carl Correns Foundation for Mathematical Biology, USA

Concerning kinetic coefficients: Often many different variants of a protein, or an enzyme, exist, with different kinetic properties. Especially in the G-protein coupled pathway, variants for adenylate cyclase, phosphodiesterase, RGS proteins, G-coupled receptors are on the order of dozens or more (including splice variants). Accordingly, the actual kinetic rate e.g. for cAMP degradation can be fine-tuned by the expression level of the different PDE's involved. These protein variants also often have differentiated expression by cellular compartment and different regulatory interactions. Still, I believe the potential for kinetic rate learning by evolutionary evolvement of enzymes into subtypes is present.

I agree that learning of a set of reactions would be a more ambitious and worthwhile project.
